# Asthma Among Children With Primary Ciliary Dyskinesia

**DOI:** 10.1001/jamanetworkopen.2024.49795

**Published:** 2024-12-09

**Authors:** Joe Zein, Arthur Owora, Hyun Jo Kim, Nadzeya Marozkina, Benjamin Gaston

**Affiliations:** 1 Department of Medicine, Division of Pulmonary and Sleep Medicine, Mayo Clinic, Scottsdale, Arizona; 2 Department of Pediatrics and Riley Hospital for Children, Indiana University School of Medicine, Indianapolis; 3 Center for Biomedical Informatics, Regenstrief Institute, Indianapolis, Indiana; 4 Department of Bioinformatics and Systems Biology, Case Western Reserve University, Cleveland, Ohio

## Abstract

This case-control study uses electronic health record data to assess the association between primary ciliary dyskinesia and asthma in children.

## Introduction

Primary ciliary dyskinesia (PCD) results from mutations in more than 50 genes affecting the structure and function of motile cilia.^1^ It is more common than previously thought, with an incidence of at least 1 in 7600 people.^2^ Asthma may be a PCD comorbidity,^3^ and antigen stasis on the airway surface in PCD may predispose patients to asthma.^4^ This study assessed whether asthma should be routinely considered as a comorbidity among children with PCD.

## Methods

This case-control study analyzed the association between PCD and asthma in children 18 years or younger in 2 large electronic health record (EHR)–based databases: the Indiana Network for Patient Care Research (INPCR) cohort of 20 million EHRs and, in a validation cohort, TriNetX, which includes 112 million EHRs. Two issues were initially addressed. First, there is no *International Statistical Classification of Diseases and Related Health Problems, Tenth Revision* (*ICD-10*) code for PCD. Second, clinicians may confuse asthma and PCD in clinical assessment and coding. Therefore, we analyzed only a combination of 2 diagnoses that are unlikely to occur together in the absence of PCD^1^: specifically, bronchiectasis (*ICD-10* code J47) and situs inversus totalis (*ICD-10* Q89.3) (B-SIT). We asked whether individuals with B-SIT also had a diagnosis of asthma (*ICD-10* code J45). We matched cases with controls (1:3 in INPCR and 1:1 in TriNetX), controlling for age, sex, ethnicity, and race. We used conditional logistic regression adjusted for the matching strategy. Race and ethnicity were self-reported by the patients (or infant or child caregiver) and adjusted for as potential confounders as part of the matching strategy. Race and ethnicity data are routinely collected in the EHR. For the INPCR, we performed a conditional exact test to compare the proportion of asthma between cases and controls; the numbers were too small to implement adjusted models. A 2-sided *P* < .05 was considered significant. This study was approved by the Indiana University and Case Western Reserve University institutional review boards, which determined that patient consent was not required because the data were deidentified. This study followed the STROBE reporting guideline.

## Results

In this study of 266 participants, including 124 B-SIT patients and 142 controls, participants were matched for age, sex, ethnicity, and race (Table 1). In the INPCR study, 9 children had B-SIT and 27 were in the control group. All 9 B-SIT patients had asthma compared with only 1 among the controls (*P* < .001). In TriNetX (matched 1:1, n = 115 each), of the 115 with B-SIT, 84 had asthma and 31 did not; in the controls, 12 had asthma and 103 did not (*P* = 2.2 × 10^−16^). The adjusted odds ratio for patients with B-SIT to have asthma was 22.3 (95% CI, 10.8-45.9; *P* < .001) (Table 2).

**Table 1.** Characteristics of the Study Participants

Characteristic	No. (%) of participants^a^	
	Cases	Controls
**Indiana Network for Patient Care Research**		
No. of individuals	9	27
Age, mean (SD), y	10.6 (5.1)	10.6 (4.9)
Sex		
Female	5 (56)	15 (56)
Male	4 (44)	12 (44)
Race		
Black	2 (22)	6 (22)
White	7 (78)	21 (78)
Ethnicity		
Non-Hispanic	6 (67)	18 (67)
Hispanic	3 (33)	9 (33)
**TriNetX**		
No. of individuals	115	115
Age, mean (SD), y	13.5 (3.4)	13.5 (3.4)
Sex		
Female	48 (42)	48 (42)
Male	67 (58)	67 (58)
Race		
Black	10 (9)	10 (9)
White	70 (61)	69 (60)
Ethnicity		
Non-Hispanic	77 (67)	77 (67)
Hispanic	38 (33)	38 (33)

^a^ Unless otherwise indicated.

**Table 2.** Asthma Among Patients With Likely Primary Ciliary Dyskinesia Compared With Age-, Sex-, Race-, and Ethnicity-Matched Controls

Characteristic	Case	Controls	*P* value
**Indiana Network for Patient Care Research**			
No. of individuals	9	27	NA
Asthma			
No	0	26 (96)	<.001
Yes	9 (100)	1 (4)	
**TriNetX**			
No. of individuals	115	115	NA
Asthma			
No	31	103	2.2 × 10^−16^
Yes	84	12	

Abbreviation: NA, not applicable.

## Discussion

This study found that most children with likely PCD (B-SIT) had asthma. This finding is consistent with a recent retrospective study^3^ and in vitro data showing that antigen stasis promotes asthma-like inflammation in the PCD airway.^4^ The fact that children with PCD characteristically have chronic, daily rhinitis^1^ does not exclude the possibility that they could also have allergic rhinitis. However, children with PCD have low nasal nitric oxide concentrations,^1,4^ whereas these concentrations are generally high in children with allergic asthma and rhinitis.^5^ Local antigen stasis in PCD could contribute to the asthma phenotype in PCD^4^ or even to asthma among patients heterozygous for PCD genes.^6^ Additional studies are needed to understand the underlying immunology. Furthermore, this study will need to be validated prospectively using formal airway reactivity testing, after drug withholding, in patients with PCD as standardized in asthma research.^5^ In summary, children with PCD may need to be evaluated for asthma. Consideration should also be given to whether some children with asthma may have cardinal features of PCD.^1^

## Notes

Supplement.
Data Sharing Statement
