# Supplementary material for: Asthma Among Children With Primary Ciliary Dyskinesia
Source: JAMA Netw Open. 2024 Dec 9;7(12):e2449795. doi: 10.1001/jamanetworkopen.2024.49795 (PMC11629127; doi:10.1001/jamanetworkopen.2024.49795)
Supplement: Supplement. — Data Sharing Statement [file jamanetwopen-e2449795-s001.pdf]

Data Sharing Statement

Zein. Asthma Among Children With Primary Ciliary Dyskinesia. *JAMA Netw Open*. Published December 09, 2024. doi:10.1001/jamanetworkopen.2024.49795

Data
Data available: No
